# Hyperthermic Isolated Limb Perfusion with TNF α and Cisplatin in the Treatment of Osteosarcoma of the Extremities: A Feasibility Study in Healthy Dogs

**DOI:** 10.1080/13577149977703

**Published:** 1999-09

**Authors:** Robert J. Van Ginkel, Charles L. H. Van Berlo, Peter C. Baas, Heimen Schraffordt Koops, Ries Van Groningen B. Stuling, Jan Elstrodt, Harald J. Hoekstra

**Affiliations:** 1 Department of Surgical Oncology Division of Surgical Oncology Groningen University Hospital PO Box 30.001 Groningen 9700 RB The Netherlands; 2 Central Animal Laboratory Groningen University Hospital Groningen The Netherlands

## Abstract

*Purpose.* The feasibility of hyperthermic isolated limb perfusion (HILP) with tumor necrosis factor-α (TNFα ) and cisplatin for the management of osteosarcoma was studied in the canine model.

*Methods.* During seven perfusions in six healthy mongrel dogs (weight 32±2 kg) technical aspects of HILP under mild hyperthermia (39– 40℃) were studied. In five experiments HILP was performed with TNFα alone (0.5 mg/l extremity volume), and in two experiments TNFα was combined with cisplatin (25 mg/l extremity volume). During the perfusions physiological parameters were monitored and TNFα and total cisplatin concentrations were determined.

*Results.* Perfusion conditions (pH, PCO_2_ , PO_2_, flow and pressure) remained within physiological ranges.Three dogs died within 24 h despite a sublethal systemic concentration of TNFα that leaked from the perfusion circuit. Three dogs were terminated; one dog after the second experiment in accordance with Dutch ethical rules; one dog showed an invagination of the small bowel resulting in an ileus; one dog because of necrosis of the perfused limb.

*Conclusions.* This feasibility study in healthy dogs demonstrated that HILP with TNFα and cisplatin was associated with a high mortality rate and does not allow us to treat dogs with spontaneous osteosarcoma with TNFα and cisplatin HILP. Therefore, an alternative model should be used in the search for the ideal combination of perfusion agents for limb sparing treatment in human osteosarcoma.


*Sarcoma* (1999) **3**, 89–94

ORIGINAL ARTICLE

### Hyperthermic isolated limb perfusion with TNFα and cisplatin in the treatment of osteosarcoma of the extremities: a feasibility study in healthy dogs

ROBERT J. VAN GINKEL,[1] CHARLES L.H. VAN BERLO,[1] PETER C. BAAS,[1] HEIMEN SCHRAFFORDT KOOPS,[1] RIES VAN GRONINGEN B. STULING,[1] JAN ELSTRODT[2] & HARALD J. HOEKSTRA[1]

*Department of Surgical Oncology[1] and Central Animal Laboratory[2], Groningen University Hospital, Groningen, The Netherlands*

**Abstract**
*Purpose.* The feasibility of hyperthermic isolated limb perfusion (HILP) with tumor necrosis factor-α (TNFα) and cisplatin for the management of osteosarcoma was studied in the canine model.
*Methods.* During seven perfusions in six healthy mongrel dogs (weight 32±2 kg) technical aspects of HILP under mild hyperthermia (39–40°C) were studied. In five experiments HILP was performed with TNFα alone (0.5 mg/l extremity volume), and in two experiments TNFα was combined with cisplatin (25 mg/l extremity volume). During the perfusions physiological parameters were monitored and TNFα and total cisplatin concentrations were determined.
*Results.* Perfusion conditions (pH, $PCO_2$, $PO_2$, flow and pressure) remained within physiological ranges. Three dogs died within 24 h despite a sublethal systemic concentration of TNFα that leaked from the perfusion circuit. Three dogs were terminated; one dog after the second experiment in accordance with Dutch ethical rules; one dog showed an invagination of the small bowel resulting in an ileus; one dog because of necrosis of the perfused limb.
*Conclusions.* This feasibility study in healthy dogs demonstrated that HILP with TNFα and cisplatin was associated with a high mortality rate and does not allow us to treat dogs with spontaneous osteosarcoma with TNFα and cisplatin HILP. Therefore, an alternative model should be used in the search for the ideal combination of perfusion agents for limb sparing treatment in human osteosarcoma.

**Key words**: *Osteosarcoma of the extremity, hyperthermic isolation, limb perfusion, chemotherapy, combination therapy, dogs*

### Introduction

Osteosarcoma is the most frequent primary malignant bone tumor in humans. Until the 1970s the most common approach to the management of localized osteosarcoma was surgical resection, amputation or radiation therapy.[1] During the last decades a definite role for neoadjuvant high-dose methotrexate and cisplatin-based polychemotherapy was established.[1–4] The potential local tumor effect of systemically administered cisplatin, however, is limited due to the nephrotoxicity and ototoxicity of cisplatin. Therefore an attempt was made to increase the local effect of cisplatin without increasing systemic toxicity by using hyperthermic isolated regional limb perfusion (HILP) with cisplatin in dogs with spontaneous osteosarcoma.[5] These studies showed an acceptable locoregional toxicity, improved functional outcome at 6 and 12 weeks, and a steadily improving radiological picture. However, the histological results were modest, with none of the dogs showing a complete response at 6 weeks after perfusion. The same experience was found in patients with sarcomas of soft tissue and bone treated with cisplatin HILP.[6] Results of recent publications and of our own experience with a new perfusion modality, which combines tumor necrosis factor-α (TNFα) and melphalan in patients with recurrent melanoma or soft tissue sarcoma, are very promising.[7,8] However, in six of eight evaluable patients with unresectable osteosarcoma of the lower limb treated with TNFα and melphalan HILP, histological evaluation revealed moderate results with ≥ 80% necrosis in three patients, 50–60% necrosis in two patients and < 50% necrosis in one patient. After TNFα and melphalan HILP, limb sparing surgery was possible in six patients.[9] As cisplatin is one of the most active chemotherapeutics in the treatment of osteosarcoma, it seems worthwhile to investigate the results of HILP with TNFα and cisplatin. With the

Correspondence to: R.J. van Ginkel, Department of Surgery, Division of Surgical Oncology, Groningen University Hospital, PO Box 30.001, 9700 RB Groningen, The Netherlands. Tel.: +31 503 612317; Fax: +31 50 3614873; email: vanginkel@compuserve.com

1357-714X print/1369-1643 online/99/020089-06 © 1999 Taylor & Francis Ltd high frequency of occurrence in dogs, canine osteosarcoma is a useful model for evaluation of new treatment regimens in humans as rapid case accrual and rapid time to reach measurable end points are possible.[10] The canine osteosarcoma therefore appears to be a valid model for studying the potential treatment of HILP with TNFα and cisplatin in the local treatment of osteosarcoma of the extremity in humans. To establish optimal HILP conditions using TNFα and cisplatin for local tumor control in dogs bearing osteosarcoma, a feasibility study in healthy dogs was undertaken.

### Materials and methods

### *Dogs*

During seven experiments in six healthy mongrel dogs with a mean average weight of 32 ± 2 kg and a mean age of 6 ± 1 years, different aspects of HILP with TNFα and cisplatin were studied. Preoperatively, all dogs were thoroughly clinically evaluated at the Central Animal Facility of the University of Groningen. The study was approved by the Animal Welfare Committee of the Faculty of Medicine of the Groningen University.

### *Anaesthetics*

The dogs fastened for 12 h and were anaesthetized with thiopental (30 mg/kg body wt., i.v.) (Pentothal, Abbott, Amstelveen, The Netherlands) and, after muscle relaxation with pancuroniumbromide (0.08 mg/kg body wt., i.v.) (Pavulon, Organon, Oss, The Netherlands), the dogs were ventilated (Ohmeda Modulus 2) with a mixture of $O_2$ and isoflurane. The oxygen concentration in the gas mixture was continuously measured by means of an oxygen analyzer (Ohmeda Modulus 2) and minute volumes (4–6 l/min) were adjusted to maintain an end-expiratory $CO_2$ concentration of 4–5% (Siemens $CO_2$ analyzer 930). The dogs were placed in the supine position on a heated mattress to maintain their normal body temperature of 38°C.[11] During the operations all dogs were given about 2 l of glucose 5% via a cephalic or internal jugular vein. Central arterial pressure was recorded as well as an ECG and diuresis.

### *Operation and perfusion techniques*

During anaesthesia the volume of the extremity was measured using Archimedes' rule (1.7–2 l). The iliac vessels were exposed under sterile conditions and collateral vessels were clipped. Cannulas were inserted into the artery (Bardic, 14–18 F) and vein (Bardic, 14–18 F). Both cannulas were connected to an extracorporeal circuit consisting of an occlusive roller pump, a cardiotomy reservoir and a bubble oxygenator with heat-exchanger. A tourniquet made of nylon was placed around the base of the extremity, using a pin in the bone and a bandage around the middle to complete the isolation of the limb from the systemic circulation. The perfusate consisted of 350 ml 5% dextran 40 in glucose 5% (Isodex, Pharmacia AB, Uppsala, Sweden), 250 ml red blood cells (canine blood donors), 250 ml plasma, 30 ml sodium bicarbonate 8.4% and 0.5 ml 5000 IU/ml heparin (Thromboliquine, Organon B.V., Oss, The Netherlands). The mixture of oxygen, air and carbon dioxide through the oxygenator was adjusted to maintain the blood gas values within the physiological range and, when necessary, bicarbonate was added to adjust the pH value.

All perfusions were performed under mild hyperthermic conditions (39–40°C) and optimal physiological conditions.[12,13] Thermistor probes (Electrolaboriet, Copenhagen, Denmark) were inserted into the subcutaneous tissues and into a muscle of the thigh just above the knee for continuous monitoring of the temperatures during perfusion. In the first five experiments TNFα was the sole perfusion agent, in the last two experiments TNFα was combined with cisplatin. The dosage of TNFα (0.5 mg/l extremity volume) (Boehringer, Ingelheim, Germany) was calculated in order not to exceed ten times the acceptable systemic levels (systemic, 10 μg/kg body wt).[14] The dosage of cisplatin (20 mg/l extremity volume) (Platinol 0.5 mg/ml, Bristol Myers Squibb, Weesp, The Netherlands) used in the perfusion had been established in a previous study and was based on a maximum tolerable dose of 30 mg/l extremity volume.[15] Cisplatin was added to the circulated perfusate in 10 min. During perfusion, serum TNFα and total cisplatin levels were determined in the regional and systemic circulation at 0, 5, 15, 30, 45, 60, 75 and 90 min by ELISA and flameless atomic absorption spectophotometry (FAAS), respectively. The perfusion time was 1 h, followed by wash-out of the extremity with 3 l of Isodex. Tourniquet, cannulas and clips were then removed and the incisions in the vessels repaired. Protamine hydrochloride (Hoffman La Roche, Mijdrecht, The Netherlands) was administered, to neutralize heparin, in a ratio of 1:1 to the initial dose of heparin. All dogs were closely observed for at least 24 h. No anti-inflammatory or analgesic drugs were administered during follow-up.

All dogs were followed for local and systemic side effects of TNFα and cisplatin perfusion, as well as survival.

### Results

Table 1 shows the characteristics of the seven experiments in six dogs. During the experiments conditions for perfusions (pH, $PCO_2$, $PO_2$) were kept within the physiological ranges, as in human perfusions. Figure 1 shows the flow, blood pressure, perfusion pressures, weight gain or loss of the extracorporeal circuit and temperature during 60 min of perfusion in the

**Table 1**. *Characteristics for the seven experiments in six dogs*

| Experiment | Body weight (kg) | Limb volume (l) | TNFα dose (mg) | Cisplatin dose (mg) | Leakage (%) | Limb toxicity | Follow-up |
|---|---|---|---|---|---|---|---|
| 1 | 35 | 2.7 | 1.3 | 0 | 8.7 | II | Dead < 24 h |
| 2 | 31.5 | 1.4 | 0.6 | 0 | 0.3 | II | Dead < 24 h |
| 3 | 26.5 | 2.3 | 1.15 | 0 | 5.1 | II | Ileus, terminated < 1 week |
| 4 | 33.5 | 2.3 | 1.15 | 0 | 4.9 | I | Alive, experiment 1 |
| 5 | 31 | 1.9 | 1 | 0 | 6.1 | n.a. | Terminated, experiment 2 |
| 6 | 29.5 | 2.0 | 1 | 50 | 33.0 | V | Necrotic limb, terminated < 1 week |
| 7 | 28.5 | 1.8 | 0.8 | 50 | 10.8 | II | Dead < 24 h |

n.a., not applicable; dog 4 underwent two experiments; limb toxicity according to Wieberdink *et al.*[26]; Grade I, no reaction, objectively and subjectively; Grade II, slight erythema, oedema or loss of sensation; Grade III, considerable erythema or oedema with some blistering, slight functional disturbances; Grade IV, extreme epidermiolysis and/or obvious damage to the deep tissues causing definite functional disturbances; Grade V, reaction that might necessitate amputation.

seven experiments. In the first five experiments, only TNFα was administered to the perfusion circuit. In the last two experiments cisplatin was added. Figure 2 illustrates the TNFα concentrations (mean ± SEM) in the perfused limb as well as in the systemic circulation of the dog during perfusion and afterwards. Peak TNFα concentrations in the perfused limb were 650 ± 158 ng/ml, and in the systemic circulation of the dog they were 37 ± 15 ng/ml. The peak systemic concentrations in the dog were in the same range as those of TNFα and melphalan HILP used in the treatment of humans at our institute.[16] Figure 3 shows the measured total cisplatin values in the last two experiments. During the experiments we were not able to perform any leakage monitoring by means of radionuclear detection techniques which are used in the clinical perfusion setting. Therefore leakage was calculated afterwards according to Stehlin with the amount of blood in the dogs estimated at 69 ml/kg body weight.[17] Calculated leakage values are summarized in Table 1.

Three dogs died within 24 h: the first two during the TNFα experiment; the third after TNFα and cisplatin perfusion. Postmortem examination of these animals did not provide any macroscopic or microscopic evidence to explain the cause of death. Three dogs were terminated; two due to treatment complications. One dog showed an invagination of the small bowel, resulting in an ileus and another was terminated 1 week after TNFα and cisplatin perfusion because of necrosis of the perfused limb. The third dog was terminated after the second experiment in accordance with Dutch ethical rules.

### Discussion

In the treatment of osteogenic sarcoma a distinction can be made between systemic therapy and locoregional treatment. High-dose methotrexate-based systemic chemotherapy is primarily administered in order to eradicate possible micrometastatic disease, and its use was a major breakthrough in the clinical treatment of osteosarcomas in the 1970s.[1,2] Today, about 60% of patients with resectable primary tumors and no metastases at the time of the initial diagnosis will be cured.[1]

The primary objective in locoregional treatment is to prevent local recurrence and allow limb salvage procedures in an attempt to preserve limb function. New surgical techniques and the development of endoprosthetic materials, coupled with systemic neoadjuvant chemotherapy, have offered less radical surgery for 40–80% of patients with osteosarcoma since the 1980s.[1,18] Procedures that increase tumor necrosis of the primary tumor, with reduction of viable tumor cells and tumor volume, could contribute to limb preservation strategies. Since its first use, cisplatin has been one of the most effective chemotherapeutic agents and has been incorporated in most systemic treatment regimens for osteosarcoma. A recent attempt to overcome its nephrotoxic and ototoxic limitations by administering cisplatin in HILP in the treatment of spontaneous canine osteosarcoma was histologically modest.[5] Promising results of recent publications and our own experience with a new combination perfusion modality (TNFα and melphalan) for recurrent melanoma or soft tissue sarcoma, but moderate histological results in patients with osteosarcoma, prompted us to investigate the combination of TNFα and cisplatin in HILP for osteosarcoma.[7–9] Since endothelial cells are supposed to play a key role in the working mechanism of TNF, osteosarcomas with a high extent of tumor vessels are of particular interest.

Before application of TNFα and cisplatin HILP in humans and client-owned osteosarcoma-bearing dogs, the present feasibility study was performed in normal healthy dogs. Despite sufficient experience in HILP in dogs as well as in humans, an unexpected high mortality rate was encountered. Although there was no mortality related to the operation, three dogs died within 24 h after perfusion (50%). This direct postoperative mortality could not be explained by a surplus of systemical leakage of TNF. In the experiment, the dog with the highest leakage and, as a

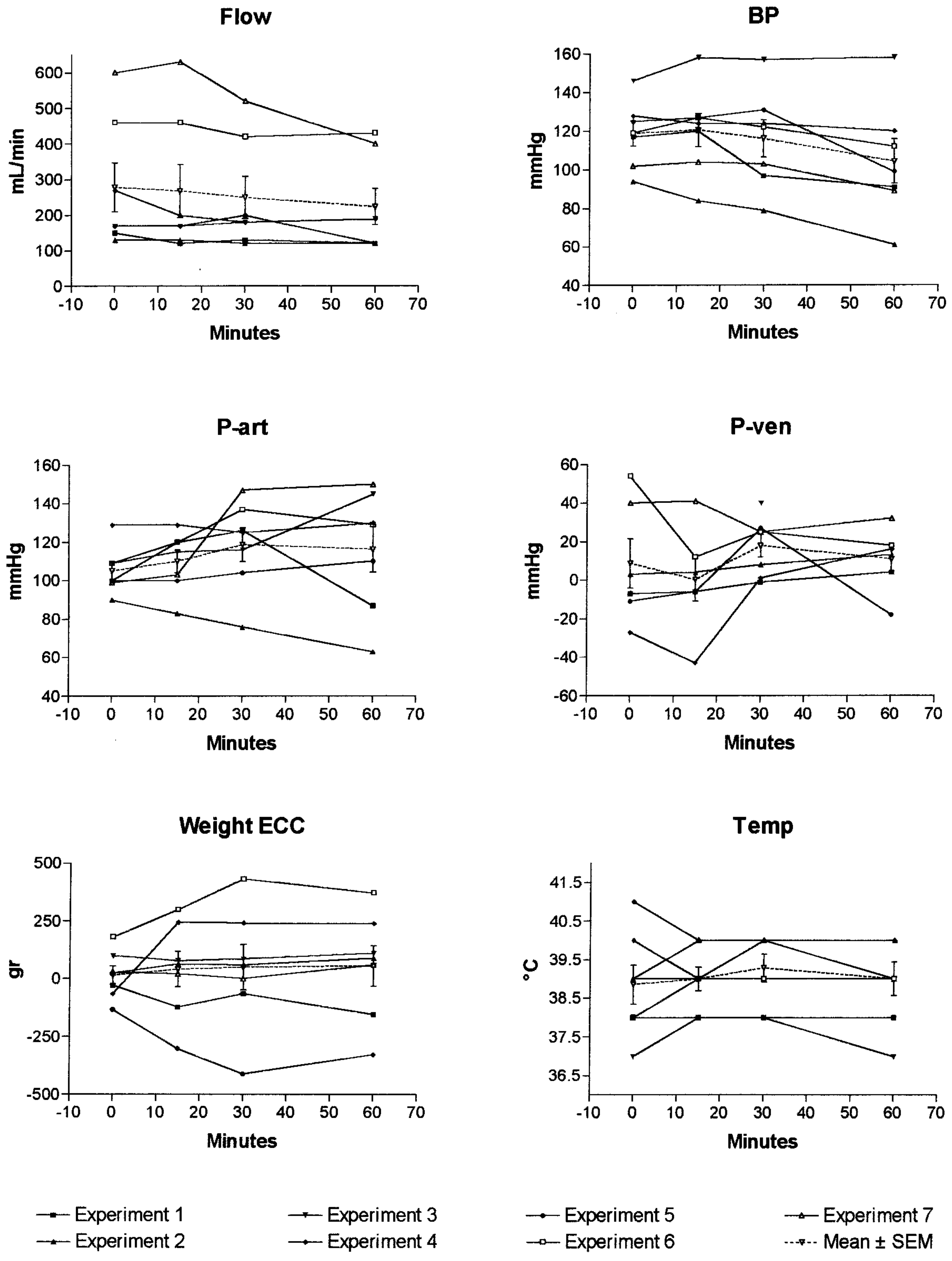

**Fig. 1.** *Perfusion characteristics (flow, systemic blood pressure of the dog (BP); arterial catheter pressure (P-art); venous catheter pressure (P-ven); extra-corporeal circulation (ECC); weight gain (+) or loss (–) and temperature of the perfused limb (°C)) in time during 60 min of perfusion in seven experiments.*

consequence, the highest systemic TNFα concentrations, survived immediately postoperatively, and the dog with the lowest leakage (lowest systemic TNFα concentrations) died within 24 h after perfusion. No correlation between leakage and mortality rate could be established. Maximal leakage encountered in these experiments was 33%, this corresponds to 330 μg TNFα given systemically per dog; since the average dog weighs 33 kg, the dose of TNFα that reaches the systemic circulation of the dog is sublethal (10 μg/kg).[14] Although only sublethal doses of TNFα leaked to the systemical circulation, the clinical picture resembled responses observed with lethal doses (>100 μg/kg), characterized by progressive

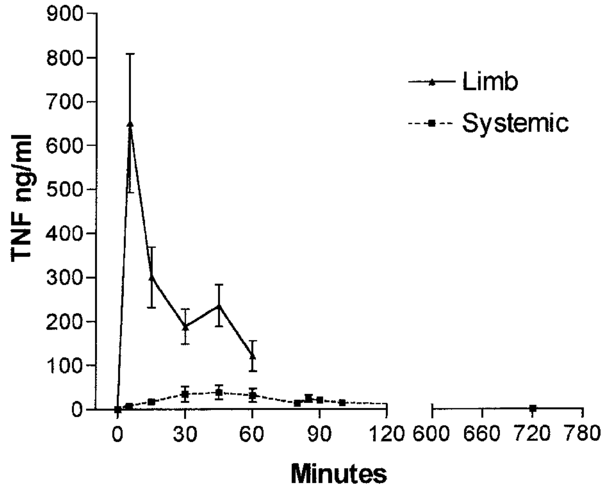

**Fig. 2.** *TNFα levels in ng/ml (mean ± SEM) as obtained by human TNFα ELISA in the perfused limb, as well as in the systemic circulation of the dog.*

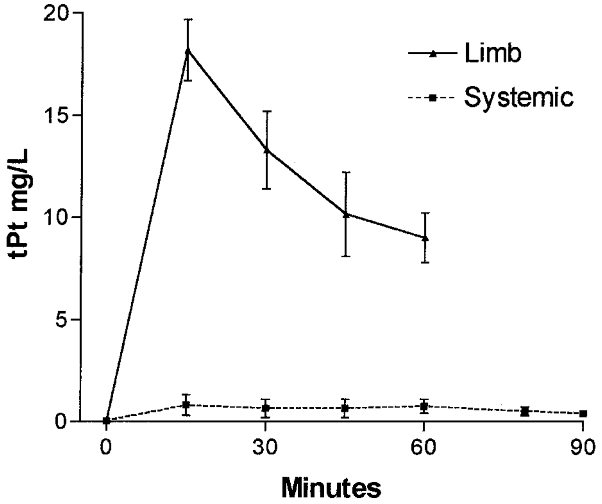

**Fig. 3.** *Total cisplatin levels (mg/l) as obtained by flameless atomic absorption spectrophotometry (FAAS) in the perfused limb as well as in the systemic circulation of the dog.*

hypotension, shock and death within 24 h.[19] Due to the lack of facilities, we were not able to support the dogs with intensive postoperative care, as is the case after human TNFα HILP. In part this could explain the observed direct postoperative mortality and supports the need for intensive treatment after TNFα HILP in the dog.

Three dogs survived the first days after perfusion; however, one dog developed an ileus and was terminated within 1 week after perfusion. One dog that underwent two experiments survived the first without morbid effects, but was terminated after the second experiment according to Dutch ethical rules. Leg toxicity consisted in slight erythema and edema in all dogs except one in the cisplatin-treated group. In this dog, necrosis of the perfused limb was encountered, necessitating termination. We have never observed necrosis of the perfused limb with the cisplatin dose used (25 mg/l extremity volume) in experiments where cisplatin was the sole perfusion agent.[15] This observation may indicate that TNFα might enhance the effect of cisplatin to the local tissues of the perfused limb. The *in vitro* anticancer potential, and overcoming cisplatin resistance with the combination of TNFα and cisplatin in different cell lines, has been established by others.[20–22] Buell *et al.* demonstrated an increased cellular cisplatin accumulation and DNA adduct formation as the possible cellular basis for the augmented cisplatin cytotoxicity in the presence of TNF and hyperthermia.[23] Recently, Anda *et al.* demonstrated that TNFα selectively promoted the *in vitro* permeability of the blood–brain barrier to CDDP without disrupting the tight junctions.[24] An improved penetration of cisplatin into the interstitial space due to a higher permeability of the vascular wall, combined with an increased cellular cisplatin accumulation and DNA adduct formation, could explain the observed necrosis of the limb in this *in vivo* model with the cisplatin dose used, which was previously non-toxic.

The observed mortality and morbidity that we encountered in this canine study was similar to the experience of Withrow and colleagues (unpublished observations). The present results in normal elderly mongrel dogs indicate that treatment of dogs with spontaneous osteosarcoma using TNFα and cisplatin HILP is not appropriate. Future research could focus on postoperative monitoring and care in dogs after TNFα HILP; perhaps a better alternative for testing the effect of TNFα with cisplatin HILP is the use of the rat osteosarcoma model described by Manusama *et al.*,[25] since rats are much less susceptible to TNFα than dogs.

### Acknowledgments

The advice and technical assistance, in the Groningen experimental TNFα and cisplatin perfusion study for spontaneous canine osteosarcoma, of A.P. Gaaikema, W.A. Buurman PhD, W.M. Molenaar MD PhD, D.R.A. Uges PhD, E.G.E. de Vries MD PhD, J. de Vries MD PhD and M.M. Weggemand-Reuvers are very much appreciated. This study was supported by a grant from the Groningen Surgical Department.

### References

1 Ham SJ, Schraffordt Koops H, van der Graaf WT, *et al.* Historical, current and future aspects of osteosarcoma treatment. *Eur J Surg Oncol* 1998; 24:584–600.
2 Rosen G, Tan C, Sanmaneechai A, *et al.* The rationale for multiple drug chemotherapy in the treatment of osteogenic sarcoma. *Cancer* 1975; 35:936–45.
3 Link MP, Goorin AM, Miser AW, *et al.* The effect of adjuvant chemotherapy on relapse free survival in patient with osteosarcoma of an extremity. *New Eng J med* 1986; 314:1600–6.
4 Eilber F, Giuliano A, Eckardt J, *et al.* Adjuvant chemotherapy for osteosarcoma: a randomized prospective trial. *J Clin Oncol* 1987; 5:21–6.
5 van Ginkel RJ, Hoekstra HJ, Meutstege FJ, *et al.* Hyperthermic isolated regional perfusion with cisplatin in the local treatment of spontaneous canine osteosarcoma: assessment of short-term effects. *J Surg Oncol* 1995; 59:169–76.

6 van Ginkel RJ, Schraffordt Koops H, de Vries EG, *et al.* Hyperthermic isolated limb perfusion with cisplatin in four patients with sarcomas of soft tissue and bone. *Eur J Surg Oncol* 1996; 22:528–31.
7 Lienard D, Ewalenko P, Delmotte JJ, *et al.* High-dose recombinant tumor necrosis factor alpha in combination with interferon gamma and melphalan in isolation perfusion of the limbs for melanoma and sarcoma. *j Clin Oncol* 1992; 10:52–60.
8 Eggermont AMM, Schraffordt Koops H, Lienard D, *et al.* Isolated limb perfusion with high-dose tumor necrosis factor-alfa in combination with interferon-gamma and melphalan for nonresectable extremity soft tissue sarcomas: a multicenter trial. *J Clin Oncol* 1996; 14:2653–65.
9 Bickels J, Manusama ER, Gutman M, *et al.* Isolated limb perfusion with tumor necrosis factor alpha and melphalan for unresectable bone sarcomas of the lower extremity. In: Manusama ER, editor. *TNFα-based isolated limb perfusion in the rat.* The Hague: Pasmans, 1998:105–17.
10 Withrow SJ, Powers BE, Straw RC, Wilkins RM. Comparative aspects of osteosarcoma. Dog versus man. *Clin Orthop* 1991; 159–68.
11 Thrall DE, Page RL, Dewhirst MW, *et al.* Temperature measurements in normal and tumor tissue in dogs undergoing whole body hyperthermia. *Cancer Res* 1986; 46:6229–35.
12 Fontijne WP, Mook PH, Elstrodt JM, *et al.* Isolated hindlimb perfusion in dogs: the effect of perfusion pressures on the oxygen supply (ptO2 histogram) to the skeletal muscle. *Surgery* 1985; 97:278–84.
13 Fontijne WP, De Vries J, Mook PH, *et al.* Improved tissue perfusion during pressure regulated hyperthermic regional isolated perfusion in dogs. *J Surg Oncol* 1984; 26:69–76.
14 Tracey KJ, Lowry SF, Fahey TJ 3d, *et al.* Cachectin/tumor necrosis factor induces lethal shock and stress hormone responses in the dog. *Surg Gynecol Obstet* 1987; 164:415–22.
15 De Vries J, Hartel RM, Schraffordt Koops H, Oosterhuis JW. Dosage of cisplatin in hyperthermic isolated regional perfusion. *Surg Res Commun* 1987; 2:107–12.
16 Zwaveling JH, Maring JK, Clarke FL, *et al.* High plasma tumor necrosis factor (TNF)-alpha concentrations and a sepsis-like syndrome in patients undergoing hyperthermic isolated limb perfusion with recombinant TNF-alpha, interferon-gamma, and melphalan. *Crit Care Med* 1996; 24:765–70.
17 Stehlin JS, Clark RL, White EC, *et al.* The leakage factor in regional perfusion with chemotherapeutic agents. *American Medical Association Arch Surg* 1960; 80:934–45.
18 Meyer WH, Malawer MM. Osteosarcoma. Clinical features and evolving surgical and chemotherapeutic strategies. *Pediatr Clin North Am* 1991; 38:317–48.
19 Eichenholz PW, Eichacker PQ, Hoffman WD, *et al.* Tumor necrosis factor challenges in canines: patterns of cardiovascular dysfunction. *Am J Physiol* 1992; 263:H668–75.
20 Mutch DG, Powell CB, Kao MS, Collins JL. In vitro analysis of the antiancer potential of tumor necrosis factor in combination with cisplatin. *Gynecol Oncol* 1989; 34:328–33.
21 Mizutani Y, Bonavida B, Overcoming cis-diammine-dichloroplatinum (II) resistance of human ovarian tumor cells by combination treatment with cis-diamminedichloroplatinum (II) and tumor necrosis factor-alpha. *Cancer* 1993; 72:809–18.
22 Sleijfer S, Le TK, de Jong S, *et al.* Combined cytotoxic effects of tumor necrosis factor-alpha with various cytotoxic agents in tumor cell lines that are drug resistant due to mutated p53. *J Immunother* 1999; 22:48–53.
23 Buell JF, Reed E, Lee KB, *et al.* Synergistic effect and possible mechanisms of tumor necrosis factor and cisplatin cytotoxicity under moderate hypoerthermia against gastric cancer cells. *Ann Surg Oncol* 1997; 4:141–8.
24 Anda T, Yamashita H, Khalid H, *et al.* Effect of tumor necrosis factor-alpha on the permeability of bovine brain microvessel endothelial cell monolayers. *Neurol Res* 1997; 19:369–76.
25 Manusama ER, Stavast J, Durante NM, *et al.* Isolated limb perfusion with TNF alpha and melphalan in a rat osteosarcoma model: a new anti-tumor approach. *Eur J Surg Oncol* 1996; 22:152–7.
26 Wieberdink J, Benckhuysen C, Braat RP, *et al.* Dosimetry in isolation perfusion of the limb by assessment of perfused tissue volume and grading of toxic tissue reactions. *Eur J Cancer Clin Oncol* 1982; 18:905–10.

## References

[PDF_00531] Anda T., Yamashita H., Khalid H., Tsutsumi K., Fujita H., Tokunaga Y., Shibata S. (1997). Effect of tumor necrosis factor-alpha on the permeability of bovine brain microvessel endothelial cell monolayers.. Neurol Res.

[PDF_00526] Buell J. F., Reed E., Lee K. B., Parker R. J., Venzon D. J., Amikura K., Arnold S., Fraker D. L., Alexander H. R. (1997). Synergistic effect and possible mechanisms of tumor necrosis factor and cisplatin cytotoxicity under moderate hyperthermia against gastric cancer cells.. Ann Surg Oncol.

[PDF_00462] Eggermont A. M., Schraffordt Koops H., Liénard D., Kroon B. B., van Geel A. N., Hoekstra H. J., Lejeune F. J. (1996). Isolated limb perfusion with high-dose tumor necrosis factor-alpha in combination with interferon-gamma and melphalan for nonresectable extremity soft tissue sarcomas: a multicenter trial.. J Clin Oncol.

[PDF_00509] Eichenholz P. W., Eichacker P. Q., Hoffman W. D., Banks S. M., Parrillo J. E., Danner R. L., Natanson C. (1992). Tumor necrosis factor challenges in canines: patterns of cardiovascular dysfunction.. Am J Physiol.

[PDF_00438] Eilber F., Giuliano A., Eckardt J., Patterson K., Moseley S., Goodnight J. (1987). Adjuvant chemotherapy for osteosarcoma: a randomized prospective trial.. J Clin Oncol.

[PDF_00481] Fontijne W. P., Mook P. H., Elstrodt J. M., Schraffordt Koops H., Oldhoff J., Wildevuur C. R. (1985). Isolated hindlimb perfusion in dogs: the effect of perfusion pressures on the oxygen supply (ptO2 histogram) to the skeletal muscle.. Surgery.

[PDF_00485] Fontijne W. P., de Vries J., Mook P. H., Elstrodt J. M., Oosterhuis J. W., Schraffordt Koops H., Oldhoff J., Wildevuur C. R. (1984). Improved tissue perfusion during pressure-regulated hyperthermic regional isolated perfusion in dogs.. J Surg Oncol.

[PDF_00428] Ham S. J., Schraffordt Koops H., van der Graaf W. T., van Horn J. R., Postma L., Hoekstra H. J. (1998). Historical, current and future aspects of osteosarcoma treatment.. Eur J Surg Oncol.

[PDF_00457] Lienard D., Ewalenko P., Delmotte J. J., Renard N., Lejeune F. J. (1992). High-dose recombinant tumor necrosis factor alpha in combination with interferon gamma and melphalan in isolation perfusion of the limbs for melanoma and sarcoma.. J Clin Oncol.

[PDF_00434] Link M. P., Goorin A. M., Miser A. W., Green A. A., Pratt C. B., Belasco J. B., Pritchard J., Malpas J. S., Baker A. R., Kirkpatrick J. A. (1986). The effect of adjuvant chemotherapy on relapse-free survival in patients with osteosarcoma of the extremity.. N Engl J Med.

[PDF_00535] Manusama E. R., Stavast J., Durante N. M., Marquet R. L., Eggermont A. M. (1996). Isolated limb perfusion with TNF alpha and melphalan in a rat osteosarcoma model: a new anti-tumour approach.. Eur J Surg Oncol.

[PDF_00506] Meyer W. H., Malawer M. M. (1991). Osteosarcoma. Clinical features and evolving surgical and chemotherapeutic strategies.. Pediatr Clin North Am.

[PDF_00517] Mizutani Y., Bonavida B. (1993). Overcoming cis-diamminedichloroplatinum (II) resistance of human ovarian tumor cells by combination treatment with cis-diamminedichloroplatinum (II) and tumor necrosis factor-alpha.. Cancer.

[PDF_00513] Mutch D. G., Powell C. B., Kao M. S., Collins J. L. (1989). In vitro analysis of the anticancer potential of tumor necrosis factor in combination with cisplatin.. Gynecol Oncol.

[PDF_00431] Rosen G., Tan C., Sanmaneechai A., Beattie E. J., Marcove R., Murphy M. L. (1975). The rationale for multiple drug chemotherapy in the treatment of osteogenic sarcoma.. Cancer.

[PDF_00502] STEHLIN J. S., CLARK R. L., WHITE E. C., HEALEY J. E., DEWEY W. C., BEERSTECHER S. (1960). The leakage factor in regional perfusion with chemotherapeutic agents.. Arch Surg.

[PDF_00522] Sleijfer S., Le T. K., de Jong S., Timmer-Bosscha H., Withoff S., Mulder N. H. (1999). Combined cytotoxic effects of tumor necrosis factor-alpha with various cytotoxic agents in tumor cell lines that are drug resistant due to mutated p53.. J Immunother.

[PDF_00477] Thrall D. E., Page R. L., Dewhirst M. W., Meyer R. E., Hoopes P. J., Kornegay J. N. (1986). Temperature measurements in normal and tumor tissue of dogs undergoing whole body hyperthermia.. Cancer Res.

[PDF_00489] Tracey K. J., Lowry S. F., Fahey T. J., Albert J. D., Fong Y., Hesse D., Beutler B., Manogue K. R., Calvano S., Wei H. (1987). Cachectin/tumor necrosis factor induces lethal shock and stress hormone responses in the dog.. Surg Gynecol Obstet.

[PDF_00441] Van Ginkel R. J., Hoekstra H. J., Meutstege F. J., Oosterhuis J. W., Uges D. R., Schraffordt Koops H. (1995). Hyperthermic isolated regional perfusion with cisplatin in the local treatment of spontaneous canine osteosarcoma: assessment of short-term effects.. J Surg Oncol.

[PDF_00539] Wieberdink J., Benckhuysen C., Braat R. P., van Slooten E. A., Olthuis G. A. (1982). Dosimetry in isolation perfusion of the limbs by assessment of perfused tissue volume and grading of toxic tissue reactions.. Eur J Cancer Clin Oncol.

[PDF_00474] Withrow S. J., Powers B. E., Straw R. C., Wilkins R. M. (1991). Comparative aspects of osteosarcoma. Dog versus man.. Clin Orthop Relat Res.

[PDF_00496] Zwaveling J. H., Maring J. K., Clarke F. L., van Ginkel R. J., Limburg P. C., Hoekstra H. J., Koops H. S., Girbes A. R. (1996). High plasma tumor necrosis factor (TNF)-alpha concentrations and a sepsis-like syndrome in patients undergoing hyperthermic isolated limb perfusion with recombinant TNF-alpha, interferon-gamma, and melphalan.. Crit Care Med.

[PDF_00453] van Ginkel R. J., Schraffordt Koops H., de Vries E. G., Molenaar W. M., Uges D. R., Hoekstra H. J. (1996). Hyperthermic isolated limb perfusion with cisplatin in four patients with sarcomas of soft tissue and bone.. Eur J Surg Oncol.

